# Influence of label and academic field on cancer stigma and subjective illness theories among medicine and psychology students: a cross-sectional online study

**DOI:** 10.1038/s41598-025-86245-y

**Published:** 2025-01-20

**Authors:** Mareike Rutenkröger, Sakine Agrali, Isabelle Scholl

**Affiliations:** Department of Medical Psychology, University Medical Center Hamburg-Eppendorf, Martinistraße 52, 20246 Hamburg, Germany

**Keywords:** Cancer survivorship, Stigmatization, Medical education, Health professional education, Psychology, Health care

## Abstract

The growing population of cancer survivors faces psychosocial challenges, including stigma. This study examined stigma toward “persons with cancer” and “cancer survivors” among medicine and psychology students, focusing on the impact of labeling. Additionally, the study explored these students’ subjective illness theories of cancer. We conducted a cross-sectional online survey with 186 psychology and 179 medicine students from German universities, assessing stigmatizing attitudes using a modified Social Distance Scale and subjective illness theories. Participants were randomly assigned to items using either term. Data analysis included univariate two-factorial ANOVA, Mann-Whitney-U-tests, and Kruskall-Wallis-tests. Results showed the strongest stigma for having a person with cancer/cancer survivor as a son-/daughter-in-law, and the lowest for having them as neighbors. Medicine students endorsed more stigmatizing statements with the label “cancer survivors,” while psychology students did so with the label “persons with cancer.” Subjective illness theories differed between groups. The study highlights minimal stigma endorsement among both student groups, with labeling influencing attitudes. These findings emphasize the need for educational initiatives in health professional curricula to address stigmatization and encourage compassionate patient care.

**Supplementary Information:**

The online version contains supplementary material available at 10.1038/s41598-025-86245-y.

## Introduction

In 2018, there were approximately 43.8 million cancer survivors worldwide who had been diagnosed within the previous five years^1^. By 2017, an estimated 4.65 million individuals in Germany were classified as cancer survivors, with the majority identified as long-term survivors (> 5 years after diagnosis)^2^. The number of cancer survivors in the United States is expected to increase to 26.0 million by the year 2040^3^. These statistics underline the significant impact of oncological advances that have transformed cancer into a chronic condition for many, contributing to a rapidly growing population of long-term survivors^4^.

When it comes to referring to persons with cancer, “cancer survivor” is a common term used in English-speaking countries^5^. However, research also indicates variations in the adoption of the term “cancer survivor” among affected individuals, with alternative terminologies proposed^6^. The most widely accepted definition views cancer survivorship as a journey that begins at the moment of diagnosis and continues throughout the individual’s lifetime. This perspective encompasses not only the period of treatment but also the long-term physical, emotional, and social experiences that follow, recognizing survivorship as an ongoing process^7,8^. In German-speaking countries, a suitable term for this group is lacking, with “cancer patient” or “person with cancer” being the prevailing labels and “cancer survivor” (“Krebsüberlebende:r” in German) seldom used^9^. Even in English-speaking countries, not all cancer survivors identify with the term, as some have negative associations with it^10^. Therefore, Bell et al. emphasise the importance of cultivating a deeper awareness of the language used in research, policy and practice, and of the nuanced meanings and assumptions embedded in these terms^11^. Cancer can be associated with psychosocial challenges, including emotional distress, reduced quality of life, fear of recurrence, social isolation, and stigma^12^. Stigmatization can be defined as the co-occurrence of its components - labelling, stereotyping, segregation, loss of status and discrimination^13^. Most studies focused on the evaluation of perceived stigmatization by persons with cancer themselves, revealing that higher cancer stigma was associated with male gender, low income, severe symptoms, lower NK cell subsets, greater depression and anxiety, poorer quality of life, loss of body image, low self-esteem, self-blame, low self-efficacy, social constraints, intrusive thoughts, poor cancer screening attendance, less physician empathy, ambivalence about emotional expression, and lower medical satisfaction^14^. Evidence also suggests that people believed to have contributed to their cancer (e.g. by excessive smoking) face more stigma and negative attitudes than those did not^15^. The stigmatization of persons with cancer is under-researched due to factors such as societal fears and prejudices^16^. The unclear causes of cancer and its less visible nature contribute to speculation and stigmatization^17–19^. Ernst et al.^17^ investigated stigmatizing attitudes towards persons with cancer in the German general population and found minimal support for stigmatizing statements. Predictors of stigmatizing attitudes were no contact with persons with cancer, age over 60 years and the assumption that one can protect oneself against cancer.

There is a significant gap in the literature regarding stigmatization towards persons with cancer among aspiring healthcare professionals. Past research has mainly examined stigmatizing attitudes toward conditions such as HIV or addiction among medical students^20–22^. Studying future physicians and psychologists is crucial as they are likely to interact frequently with persons with cancer when providing medical and psychosocial care.

Hence, our study aimed to fill the identified gap in literature concerning stigma towards persons with cancer, particularly examining how such stigma varies across academic disciplines and is influenced by labeling practices. The objective of the study is to answer the following research questions:

- How do medical and psychology students differ in their endorsement of stigmatizing statements based on the label used, specifically between “persons with cancer” and “cancer survivors”?
- In what ways do medical and psychology students’ subjective theories of cancer illness vary?

## Methods

### Study design and population

This study used a quantitative, cross-sectional design and employed an anonymous open online survey to collect data. This type of survey is distributed via the internet and made accessible to a broad audience. The survey was administered through the UniPark platform^23^, which provided a secure and anonymous environment for data collection. The study followed the guidelines outlined in the Checklist for Reporting Results of Internet E-Surveys (CHERRIES)^24^ (Supp. 1), ensuring rigorous and transparent reporting throughout the research process.

### Data collection

Data collection began on October 23 and ended on November 1, 2023, when a sufficient number of participants was reached. Eligible participants were undergraduate students enrolled in psychology or medicine programs at a German university, who were proficient in German. We used a convenience sampling strategy, recruiting participants through university mailing lists and social media platforms. The UniPark software automatically assigned students to one of four groups, each differentiated by academic field and by the phrasing used in questionnaires - either “cancer survivors” or “persons with cancer.” This process ensured balanced sub-sample sizes across all groups.

The survey was designed using a forced-choice format to minimize missing data. If a question was left unanswered, participants received a prompt indicating that a response was required. The survey was designed to prevent submission until all items were completed. IP address tracking was used to prevent multiple entries by denying multiple entries from the same address. The technical functionality of the survey was thoroughly tested by two members of our research team prior to its launch, ensuring that all aspects of the survey operated smoothly and reliably. The questionnaire was divided into 8 pages with a maximum of 20 items per page. Consistency or completeness checks were performed using JAVAScript. Participants were able to review and change their answers through a back button. Prior to participation, all participants provided informed consent, acknowledging the purpose and procedures of the study (duration of study, data storage) and their right to withdraw at any time. Ethical approval for the study was obtained from the Ethics Committee of the University Medical Center Hamburg-Eppendorf (approval no. LPEK-0666), in accordance with the Declaration of Helsinki and Good Scientific Practice guidelines.

### Questionnaires

#### Demographic variables

The participants’ age, gender, academic field, relationship status, and urban or rural upbringing were assessed using single-item measures.

#### Stigmatization towards persons with cancer and subjective illness theories

To record the extent of endorsement with stigmatizing statements, we used the German translation of the Social Distance Scale (SDS)^25^. This measure, initially developed by Link et al.^26^, examines diverse contexts of interaction and stigma sensitivity, encompassing professional, neighborhood, and familial aspects. The German version used in this study expands the inquiry into cancer-related stigmatization. The utilized items encompassed statements such as “I would not give a job to a person with cancer at a friend’s house”. Two additional items that were used by Ernst et al. for the measurement of structural discrimination have also been included in this study: “I believe individuals with cancer should face higher health insurance premiums” and “I would avoid using dishes that have been used by someone with cancer.” Answers were recorded on a three-point scale (“yes, agree”, “undecided” and “no, disagree”). The internal consistency of the questionnaire used by Ernst et al. has a value of Cronbach’s alpha = 0.81 across all nine items.

In accordance with Ernst et al.’s study^17^, participants ranked their subjective illness theories of cancer based on 17 key risk factors outlined by the Robert Koch Institute, encompassing factors like “lack of exercise,” “hereditary factors,” “smoking,” and “UV-radiation.” Psycho-etiological assumptions were also considered. Responses ranged from “no influence at all” (scale value 1) to “a very strong influence” (scale value 5) for each factor. Another single item that was surveyed was whether cancer was the comparatively most dangerous disease (based on the ranking of the danger of the seven diseases to be assessed: “cancer”, “jaundice”, “AIDS”, “heart attack”, “stroke”, “paraplegia”, “mental illness”).

Two groups were established in the study: “*cancer survivors*” and “*persons with cancer*.” Each participant encountered all nine statements, either with the term “*cancer survivor*” or the term “*person with cancer*”, randomized across medicine and psychology students. Depending on the randomly assigned term, subsequent examples retained the same terminology. To ensure balanced group sizes, both medicine and psychology students were randomly assigned to label groups. The manipulation aimed to uncover variations in stigmatization towards patients with cancer and to assess the impact of labeling on the extent of stigmatization.

#### Control variables

The Scale for the Assessment of Test Distortion through Positive Self-Presentation and Socially Desirable Response Tendencies (SEA)^27^ was utilized to detect biases from positive self-presentation and socially desirable responses. The scale comprises seven items, such as “I have gossiped about others or thought badly about them before.” Responses were recorded on a four-point scale ranging from “strongly disagree” to “strongly agree.” The SEA has an internal consistency of Cronbach’s alpha = 0.70.

Participants were asked whether they had previously had personal contact with persons diagnosed with cancer, with response options including: “yes, in a private setting,” “yes, in a university context,” “yes, both,” or “no, I have not had any contact.” The “private context” was specified as interactions occurring in students’ personal lives outside of academia, such as with family and friends. Conversely, the “professional context” referred to experiences encountered within their academic careers. Participants were also asked whether they had already undergone a cancer preventative screening (“no”/”once”/”several times”) and how they rated their personal ability to protect themselves against cancer on a five-level Likert scale (“very good protection options” to “not at all”).

### Data evaluation

Statistical analysis was performed using SPSS version 28.0^28^. To ensure data quality and participant attention, an attention control item was embedded in the SDR questionnaire. This item instructed participants to select a specific response (“Please check ‘3’ for this item”). Only responses that passed this attention check were included in the final data analysis.

Demographic data were analyzed descriptively to provide an overview of the study population. To explore differences in stigmatization levels between medical and psychology students based on the labeling (“person with cancer” or “cancer survivor”), a univariate two-factorial analysis of variance (ANOVA) was employed. A priori, the minimum required sample size was calculated for a univariate two-factorial analysis to detect small effect sizes, with a target of *N* = 176, using G*Power. Robustness checks were performed, including assessments of variable scaling, independence of observations, normality of distributions, and homoscedasticity of variance. The dependent variable was the sum score of the SDS. Sensitivity analysis was performed by adding the control variables (SEA score dichotomized at the cut-off of 19 and contact with persons with cancer as confounders to the ANOVA to check the robustness of the results.

Effect sizes were calculated using *η*^2^ for ANOVA results and Cohen’s d for other comparisons, following Cohen’s guidelines^29^. Normality was assessed using the Shapiro-Wilk test, which indicated that both groups were not normally distributed (*p* < .001). Therefore, nonparametric Mann-Whitney-*U*-tests were used to compare subjective illness theories between groups.

Additional group comparisons were made using Mann-Whitney-*U*-tests for binary variables (e.g., cancer diagnosis) and Kruskal-Wallis tests for categorical variables (e.g., personal experience with cancer preventative screening, and perceived personal protective ability).

For all inferential statistics, the significance threshold was set at 5% (α = 0.05). The Bonferroni-Holm correction was applied to adjust for potential inflation of type I error due to multiple testing^30^.

## Results

### Sample characteristics

A total of *N* = 492 participants started the survey with *N* = 365 students completing it (20% view rate, 74% completion rate). After data cleansing, which included filtering for attention check compliance, the final sample comprised 357 participants: 186 psychology students and 179 medical students. Psychology and medicine students were predominantly female (85.6% and 68.2%, respectively). In both groups, about half of students were raised in urban areas. Only a small percentage of participants reported a prior cancer diagnosis. The majority of psychology students (72.4%) reported having personal contact with persons with cancer solely in a private setting. Conversely, half of medical students (50.6%) indicated having interacted with persons with cancer, with exposure occurring both in private and academic contexts. Table 1 presents demographic characteristics of participants in more detail.

**Table 1:** Sample characteristics.

	Academic field	
	Psychology (*n* = 181)	Medicine (*n* = 176)
	*n* (%)	*N* (%)
Demographic variables		
Gender		
Female	155 (85.6)	120 (68.2)
Male	24 (13.3)	55 (31.3)
Other	2 (1.1)	1 (0.6)
Relationship status		
Single	73 (40.3)	81 (46.0)
In a relationship, not cohabiting	53 (29.3)	50 (28.4)
In a relationship, cohabiting	44 (24.3)	37 (21.0)
Married	8 (4.4)	6 (3.4)
Not specified	3 (1.7)	2 (1.1)
Degree		
Bachelor’s	102 (56.4)	n.a.
Master’s	77 (43.6)	n.a.
Study progress (semesters)		
1–3	110 (60.8)	33 (18.8)
4–6	55 (30.3)	47 (26.7)
7–9	16 (8.8)	56 (31.8)
> 10	n.a.	43 (24.4)
Residential area raised in		
Urban	89 (49.2)	90 (51.1)
Rural	79 (43.6)	70 (39.8)
Both	13 (7.2)	16 (9.1)
Cancer-related control variables		
Prior cancer diagnosis		
Yes	2 (1.1)	5 (2.8)
No	179 (98.9)	171 (97.2)
Contact to persons with cancer		
Yes, in private context	131 (72.4)	63 (35.8)
Yes, in academic context	8 (4.4)	17 (9.7)
Yes, in both contexts	18 (9.9)	89 (50.6)
No, not yet	24 (13.3)	7 (4.0)
Personal protection ability		
Not at all	0 (0.0)	6 (3.4)
Somewhat	37 (20.4)	38 (21.6)
Neither nor	89 (49.2)	97 (55.1)
Good	51 (28.2)	31 (17.6)
Very good	4 (2.2)	4 (2.3)

*n.a.* not applicable.

### Endorsement of stigmatizing statements

The highest level of endorsement of the presented statements is observed in the item “I would have a problem having a person with cancer/cancer survivor as a son-in-law/daughter-in-law.”, and “I would not use the same dishes that a person with cancer/cancer survivor has already used.” Conversely, the highest rejection of stigmatizing statements is observed in the item “I would be uncomfortable having a person with cancer/cancer survivor in my neighborhood.” Discriminatory practices, such as charging higher health insurance premiums or denying job opportunities to people with cancer, particularly in the “cancer survivor” group, were strongly opposed by psychology and medicine students. Further details regarding the frequencies for each individual item can be found in Table 2. Total SDS scores for psychology students ranged from 16 to 27 (M = 19.07, SD = 1.43)) in the “person with cancer” group and from 17 to 22 (M = 18.7, SD = 0.89) in the “cancer survivor” group. For medicine students, SDS scores ranged from 15 to 21 (M = 18.62, SD = 1.01) in the “person with cancer” group and from 15 to 21 (M = 18.86, SD = 0.82) in the “cancer survivor” group. The results of the ANOVA indicate that the field of study does not have a significant main effect on the extent of stigmatization (*F*^1,3^ = 1.77, *p* = .18), with the first number^1^ is the degrees of freedom for the between-group variance (the groups being compared), and the second number^3^ is the degrees of freedom for the within-group variance (the variability within the groups). Similarly, labeling does not show a significant main effect on the degree of stigmatization (*F*^1,3^ = 0.23, *p* = .63). A significant interaction effect between the two variables was observed concerning the endorsement of stigmatizing statements (*F*^1,3^ = 6.26, *p* = .01, *η*^2^ = 0.02, *R*^2^ = 0.02). Medicine students showed greater endorsement of stigmatizing statements when the label “cancer survivors” was used, whereas psychology students showed greater stigmatization when the label “persons with cancer” was used.

**Table 2:** Descriptive data for endorsement of stigmatizing statements on the SDS for medicine and psychology students in the labeling conditions.

Item	Academic field											
	Psychology						Medicine					
	Yes, I agree (%)		Undecided (%)		No, I disagree (%)		Yes, I agree (%)		Undecided (%)		No, I disagree (%)	
	PWC	CS	PWC	CS	PWC	CS	PWC	CS	PWC	CS	PWC	CS
I think that persons with cancer/cancer survivors should pay higher health insurance contributions.	0.0	2.2	5.5	2.7	94.5	94.4	0.0	1.1	1.2	2.2	98.8	96.7
I would not give a person with cancer/cancer survivor a job with a friend.	2.2	0.0	1.1	3.6	96.7	95.5	3.5	0.0	4.7	2.2	91.8	97.8
I would easily accept a person with cancer/cancer survivor into my circle of friends.	87.9	92.2	6.6	4.5	5.5	2.2	95.3	97.8	3.5	2.2	1.2	0.0
I would have a problem having a person with cancer/cancer survivor as a son-in-law/daughter-in-law.	3.3	4.2	19.8	10.8	76.9	82.2	9.4	3.3	16.5	6.6	74.1	90.1
I would trust a person with cancer/cancer survivor to take care of my child.	80.2	91.1	13.2	5.4	6.6	2.2	78.8	96.7	17.6	2.2	3.5	1.1
I would not use the same dishes that a person with cancer/cancer survivor has already used.	6.6	6.7	1.1	4.5	92.3	87.8	8.2	3.3	5.9	1.1	85.9	95.6
I would be uncomfortable having a person with cancer/cancer survivor in my neighborhood.	0.0	0.1	0.0	0.9	100.0	98.9	0.0	0.0	0.0	0.0	100.0	100.0
I would have no problem working with someone who has cancer/is a cancer survivor.	92.3	96.7	4.4	2.7	1.1	0.0	97.6	97.8	2.4	0.0	0.0	2.2
I would rent a room to a person suffering from cancer/a cancer survivor without a second thought.	92.3	98.2	6.6	1.8	1.1	0.0	100.0	97.8	0.0	2.2	0.0	0.0

For psychology students: “person with cancer” (= PWC), *n* = 91, condition “cancer survivor” (= CS), *n* = 90, for medical students: PWC *n* = 85, CS *n* = 91.

### Differences in subjective illness theories between medicine and psychology students

**Fig. 1:**
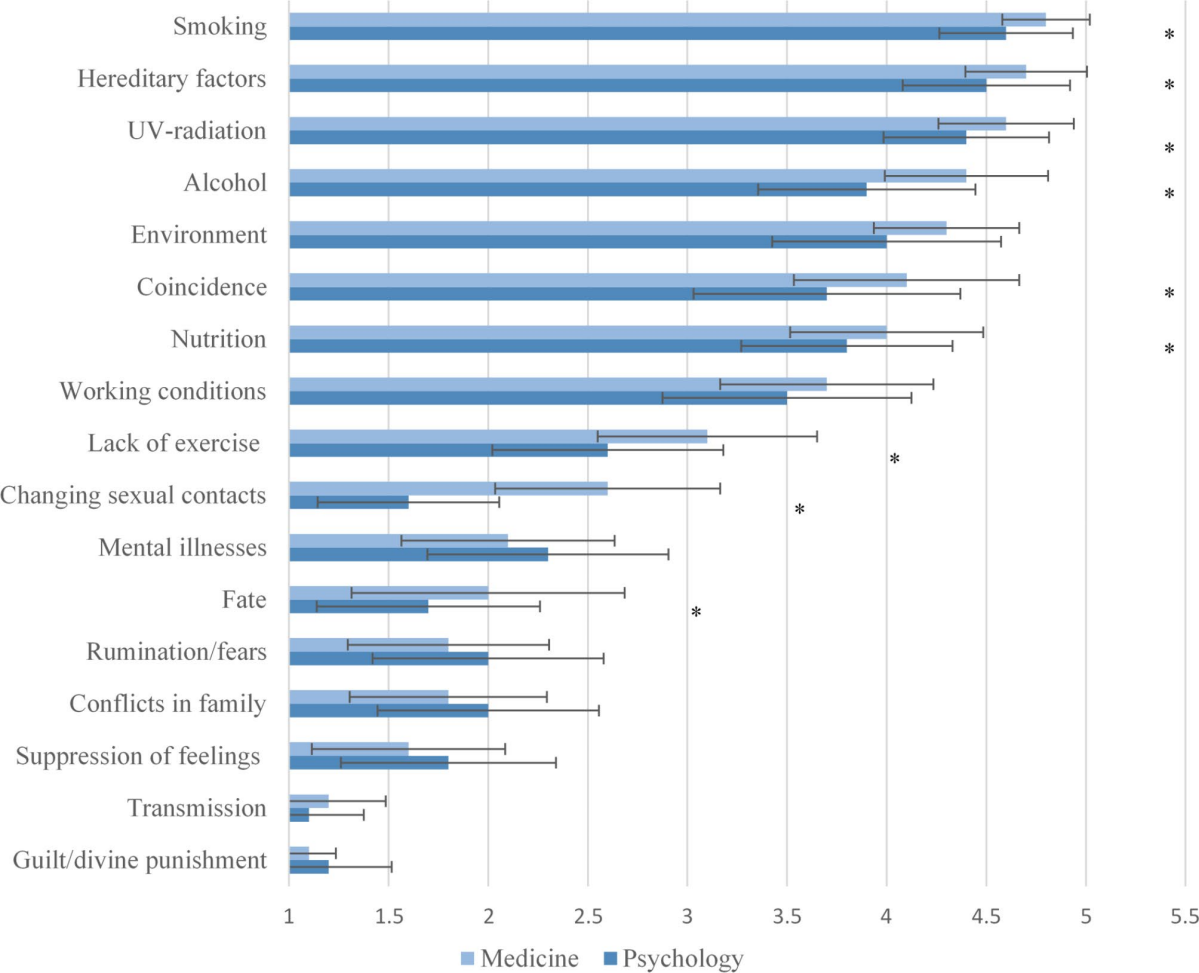
Group differences between medicine and psychology students for the subjectively perceived influence of various aspects on the development of cancer. *p < .05, Medicine students *n* = 176, psychology students *n* = 181, ranking scale from 1 (no influence) to 5 (significant influence).

Both medicine and psychology students identified hereditary factors as the most influential in cancer development (Fig. 1 and Supplementary Material 2). Psychology students rated infection from another person as least influential, whereas medicine students attributed the least influence to feelings of guilt or perceptions of divine punishment. Significant differences emerged in ratings for factors such as changing sexual partners, lack of exercise, coincidence, alcohol, UV-radiation, and smoking.

**Fig. 2:**
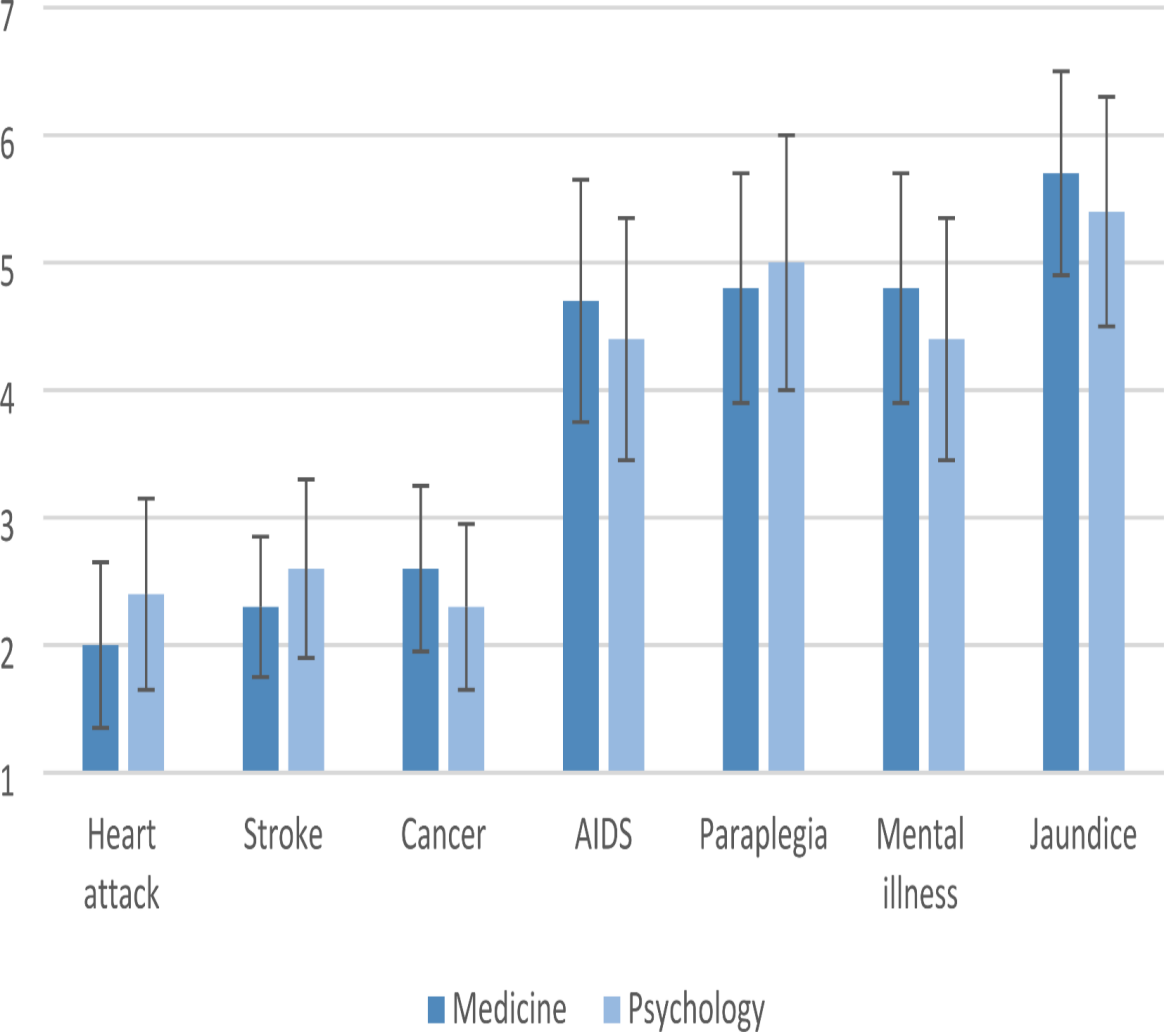
Group differences between medicine and psychology students for subjective perception of the danger of various diseases. Ranking scale from 1 (most dangerous) to 7 (least dangerous).

For the ranking of diseases, there are observable differences in the ranking trends between medicine and psychology students; however, these differences did not reach statistical significance after applying corrections for multiple testing, see Fig. 2.

### Sensitivity analysis

The data analysis highlighted that the mean value for positive self-portrayal was *M* = 19.88 (*SD* = 3.39) for psychology students and *M* = 19.31 (*SD* = 3.57) for medicine students. Including the dichotomized SEA score in the ANOVA maintained a significant interaction effect between labeling and academic field, F^1,7^ = 4.81, *p* = .046, η^2^ = 0.41, R^2^ = 0.03. However, adding the variable “contact with persons with cancer” to the model reduced the interaction effect of labeling and academic field to non-significance, F^1,15^ = 0.18, *p* = .67. Adding both variables to the model also resulted in an insignificant interaction effect, F^1,28^ = 0.10, *p* = .92.

## Discussion

The study aimed to explore variations in the endorsement of stigmatizing statements based on the labeling of individuals with cancer. A total of 357 medicine and psychology students participated, completing the SDS and items related to subjective illness theories of cancer.

Analyses examined differences in endorsement of stigmatizing statements between groups labeled as “persons with cancer” and “cancer survivors.” Overall, both medical and psychology students showed relatively low levels of stigmatization in both labeling conditions. While no significant main effects were observed for labeling conditions alone, a significant small interaction effect emerged. These findings suggest that the type of label used may differentially influence stigmatizing attitudes, with different effects depending on the field of study. However, as the effect was small, it should not be the subject of over-interpretation. On item level, notable findings include opposition to discriminating practices such as charging higher health insurance premiums or withholding job opportunities from individuals with cancer, particularly in the “cancer survivor” group. These findings underscore the importance of using sensitive terminologies and tailored support for individuals at different stages of the cancer journey. Differences in beliefs about cancer etiology were observed between medical and psychology students, with a strong effect size noted for the belief that changing sexual partners contributes to cancer risk. Moderate effects were found for the belief that alcohol consumption is a risk factor, while minimal effects were observed for factors such as lack of exercise, coincidence, UV-radiation, and smoking.

The results from the sensitivity analysis suggest that when the variable “contact with people with cancer” is added, this interaction effect becomes insignificant, indicating that personal experience with persons with cancer is likely to influence how students respond to labeling, regardless of their academic field. This suggests that contact with persons with cancer may play a moderating role, reducing the initially observed differences between psychology and medical students. In contrast, self-presentation tendencies did not influence the interaction, suggesting that personal experience, rather than general self-image, is key to understanding the relationship between labeling and academic field.

One significant challenge in addressing stigmatization is the absence of standardized terminology for cancer survivors in German-speaking contexts^5^. This lack of uniformity in labeling can contribute to misunderstandings and potentially reinforce stigmatizing attitudes. Hence, participants might have encountered difficulty in interpreting the term “cancer survivor.” Enhancing educational efforts becomes even more critical in this context. By incorporating modules on cancer awareness, empathy training, and effective communication strategies into medical and psychology curricula, future healthcare professionals can develop a deeper understanding of the challenges faced by persons with cancer. Moreover, these initiatives can foster sensitivity towards the language used to describe individuals affected by cancer, promoting more respectful and supportive interactions in clinical and non-clinical settings alike.

Lebel et al.^15^ found that individuals who are perceived to have engaged in behaviors contributing to their cancer face heightened negative attitudes and experience more severe stigma compared to those who are not perceived to have contributed to their illness. While it is important to note that our study did not analyze the specific impact of etiological misconceptions on stigma, our results showed that there is a certain proportion of medicine and psychology students who hold misconceptions about cancer etiology. Cancer-related stigmatization leads to misconceptions about persons’’ ability to control their illness through positive thinking, placing undue burden on both the affected and their families. Additionally, misconceptions about the normalcy of depression and anxiety in persons with cancer may lead clinicians and persons with cancer to overlook the potential benefits of psychosocial interventions, perpetuating stigma surrounding mental health support in cancer care^31^. Future studies should examine the potential relationship between etiological misconceptions and cancer stigma in medicine and psychology students.

Many persons with cancer encounter negative reactions from others, potentially leading to adverse health outcomes^32,33^. It’s crucial for clinicians to recognize the stigma surrounding cancer, as it profoundly affects the affected emotional well-being and behavior, ultimately impacting their quality of life. To tackle this issue effectively, it is important that curricula in medical and psychological training programs include comprehensive education on cancer etiology, emphasizing evidence-based information to dispel misconceptions and myths. By providing students with a thorough understanding of cancer, educational institutions can empower future healthcare professionals to engage with persons with cancer compassionately and without stigma or judgment. Furthermore, research suggests that improving patient-provider communication may be an effective intervention for reducing cancer stigma^34–36^.

Our study can only be compared to that of Ernst et al.^17^ to a limited extent, as our focus was on labeling in addition to comparing the study groups. In contrast, Ernst et al. investigated the endorsement of stigmatizing statements towards persons with cancer in the general population in Germany. Especially medicine students are exposed to persons with cancer from an early stage in their studies, and both academic groups are highly educated. Also, the students were, as expected, notably younger than the population in Ernst et al.’s study. This distinguishes them from the general population.

### Strength and limitations

The strength of our study lies in its diverse sample of psychology and medical students, which allows for a comprehensive examination of stigmatizing attitudes toward patients with cancer. By using random assignment and a modified social distance scale, the study effectively isolates the effects of labeling on stigma. In addition, the inclusion of control variables and the use of multiple statistical analyses provide a thorough understanding of the factors influencing these attitudes, thereby enhancing the validity and reliability of our findings.

A notable limitation of the study is its cross-sectional design and the presence of elevated levels of positive self-presentation tendencies in both medical and psychology student groups. To address this, participants with SDR scale values exceeding 20 could have been considered for exclusion. However, the correlation analysis between the SDR scale and endorsement of stigmatizing statements did not reveal a significant relationship, suggesting that despite elevated positive self-presentation tendencies, participants’ responses to stigmatization-related items were not substantially influenced. Consequently, these groups were not excluded from subsequent analyses to maintain a larger sample size. Nevertheless, acknowledging this limitation is essential.

Additionally, as Ernst et al.^17^ discussed in their work, it is important to critically evaluate the use of a 3-stage response scale (yes, no, undecided) as it may result in limited differentiation and potential measurement errors such as ceiling effects. This can also lead to a high proportion of undecided respondents.

Due to the open survey format, we do not have sufficient information on how many students our survey reached. Convenience sampling is also a limitation of our study because it may result in a non-representative sample, limiting the generalizability of the findings to a broader population. However, representativeness was not a primary goal of this study, as our focus was on exploring specific attitudes and experiences within a particular student population rather than generalizing findings to a broader population. Furthermore, it’s worth noting that our study did not examine the influence of cultural factors on cancer stigma, despite the fact that they are known to play a significant role in shaping attitudes toward illness. Cultural beliefs, values, and norms vary widely across different societies and can profoundly influence perceptions of cancer, including whether it’s viewed as a taboo subject, how individuals with cancer are treated, and what kind of support they receive^37,38^.

## Conclusion

Understanding the impact of labeling on stigmatization of persons with cancer is crucial for preparing future healthcare professionals to engage in patient-centered healthcare delivery. Therefore, research should also involve other professional groups like nursing students to inform intervention strategies for reducing cancer-related stigma in clinical settings. Seminars focusing on effective communication could enhance sensitivity among prospective physicians and psychologists, while instructional content should emphasize genuine factors contributing to cancer etiology. Our findings suggest that personal contact with persons with cancer may reduce the influence of academic background on labeling attitudes, highlighting the importance of experiential learning in training programs. Strategic dissemination of diverse content tailored for groups with frequent interaction with persons with cancer is essential for mitigating stigmatizing attitudes. Future studies could enhance external validity of our results by including a more diverse sample, considering cultural factors, and testing the findings across different contexts and populations.

## Electronic supplementary material

Below is the link to the electronic supplementary material.

Supplementary Material 1

## Data Availability

The datasets analysed during the current study are available from the corresponding author on reasonable request.

## References

[1] The Cancer Atlas [Internet]. [cited 2024 Aug 6]. Cancer Survivorship. Available from: http://canceratlas.cancer.org/rky

[2] Arndt, V., Dahm, S. & Kraywinkel, K. Krebsprävalenz in Deutschland 2017: Anzahl Der Cancer survivors basierend auf Daten bevölkerungsbezogener Krebsregister. *Onkol* **27** (8), 717–723 (2021).

[3] Bluethmann, S. M., Mariotto, A. B. & Rowland, J. H. Anticipating the silver tsunami: prevalence trajectories and Co-morbidity Burden among Older Cancer survivors in the United States. *Cancer Epidemiol. Biomarkers Prev.* **25** (7), 1029–1036 (2016). 27371756 10.1158/1055-9965.EPI-16-0133 PMC4933329

[4] Boland, L., Bennett, K. & Connolly, D. Self-management interventions for cancer survivors: a systematic review. *Support Care Cancer Off J. Multinatl Assoc. Support Care Cancer*. **26** (5), 1585–1595 (2018). 10.1007/s00520-017-3999-7 29199362

[5] Arndt, V. „Cancer survivorship in Deutschland – Epidemiologie Und Definitionen. *Forum (Genova)*. **34** (2), 158–164 (2019).

[6] Nyblade, L., Stockton, M., Travasso, S. & Krishnan, S. A qualitative exploration of cervical and breast cancer stigma in Karnataka, India. *BMC Womens Health*. **17**, 1–15 (2017). 28768506 10.1186/s12905-017-0407-x PMC5541646

[7] Marzorati, C., Riva, S. & Pravettoni, G. Who is a Cancer Survivor? A systematic review of published definitions. *J. Cancer Educ.* **32** (2), 228–237 (2017). 26854084 10.1007/s13187-016-0997-2

[8] Mullan, F. *Seasons of Survival: Reflections of a Physician with cancer*Vol. 313 (New England Journal of Medicine. Mass Medical Soc, 1985). 10.1056/NEJM198507253130421 4010738

[9] Weis, J. & Heckl, U. Psychoedukation mit Krebspatienten: Hintergrund Und Wissenschaftliche Evidenz. *Onkol* **27** (1), 54–62 (2021).

[10] Wanzer, M. B., Simon, K. G. & Cliff, N. J. Interpreting cancer survivors’ perceptions of the survivor label through social identity and communication accommodation theories. *Health Commun.* **37** (13), 1600–1608 (2022). 33823693 10.1080/10410236.2021.1909263

[11] Bell, K. & Ristovski-Slijepcevic, S. Cancer Survivorship: why Labels Matter. *J. Clin. Oncol.* **31** (4), 409–411 (2013). 23270001 10.1200/JCO.2012.43.5891

[12] Berry, L. L., Davis, S. W., Godfrey Flynn, A., Landercasper, J. & Deming, K. A. Is it time to reconsider the term cancer survivor? *J. Psychosoc Oncol.* **37** (4), 413–426 (2019). 30614422 10.1080/07347332.2018.1522411

[13] Link, B. G. & Phelan, J. C. Conceptualizing Stigma. *Annu. Rev. Sociol.* **27** (1), 363–385 (2001).

[14] Huang, Z., Yu, T., Wu, S. & Hu, A. Correlates of stigma for patients with cancer: a systematic review and meta-analysis. *Support Care Cancer*. **29** (3), 1195–1203 (2021). 32951087 10.1007/s00520-020-05780-8

[15] Lebel, S. et al. From normal response to clinical problem: definition and clinical features of fear of cancer recurrence. *Support Care Cancer*. **24**, 3265–3268 (2016). 27169703 10.1007/s00520-016-3272-5

[16] Garcia-Prieto, P. & Psycho-Oncology A Patient’s View. In: Goerling U, Mehnert A, editors. Psycho-Oncology [Internet]. Cham: Springer International Publishing; 2018 [cited 2024 Feb 14]. pp. 57–66. (Recent Results in Cancer Research; vol. 210). Available from: http://link.springer.com/10.1007/978-3-319-64310-6_4 10.1007/978-3-319-64310-6_4 28924679

[17] Ernst, J. et al. Stigmatisierende Einstellungen gegenüber Krebspatienten – Ergebnisse Einer repräsentativen Bevölkerungsbefragung. *PPmP - Psychother. · Psychosom. · Med. Psychol.* **66** (03/04), 112–119 (2016). 10.1055/s-0042-100736 27035440

[18] Balmer, C., Griffiths, F. & Dunn, J. A qualitative systematic review exploring lay understanding of cancer by adults without a cancer diagnosis. *J. Adv. Nurs.* **70** (8), 1688–1701 (2014). 25180371 10.1111/jan.12342

[19] Esser, P. et al. Body image mediates the effect of cancer-related stigmatization on depression: a new target for intervention. *Psychooncology* **27** (1), 193–198 (2018). 28685499 10.1002/pon.4494

[20] Shah, S. M., Heylen, E., Srinivasan, K., Perumpil, S. & Ekstrand, M. L. Reducing HIV stigma among nursing students: a brief intervention. *West. J. Nurs. Res.* **36** (10), 1323–1337 (2014). 24569699 10.1177/0193945914523685 PMC4459739

[21] Kingori, C., Nkansah, M. A., Haile, Z., Darlington, K. A. & Basta, T. Factors associated with HIV related stigma among college students in the Midwest. *AIMS Public. Health*. **4** (4), 347 (2017). 29546222 10.3934/publichealth.2017.4.347 PMC5690459

[22] Ma, H. & Loke, A. Y. A scoping review of an HIV/AIDS-related stigma-reduction intervention for professionals and students from health-related disciplines. *Int. J. Sex. Health*. **32** (2), 94–129 (2020).

[23] Tivian, X. I. G. H. UniPark. (2023).

[24] Eysenbach, G. Improving the quality of Web surveys: the Checklist for Reporting Results of Internet E-Surveys (CHERRIES) [Internet]. Vol. 6, Journal of medical Internet research. Gunther Eysenbach Centre for Global eHealth Innovation, Toronto, Canada; [cited 2024 Aug 6]. p. e34. (2004). Available from: https://www.jmir.org/2004/3/e34/. 10.2196/jmir.6.3.e34 PMC1550605 15471760

[25] Angermeyer, M. C. & Matschinger, H. Social distance towards the mentally ill: results of representative surveys in the Federal Republic of Germany. *Psychol. Med.* **27** (1), 131–141 (1997). 9122293 10.1017/s0033291796004205

[26] Link, B. G., Cullen, F. T., Frank, J. & Wozniak, J. F. The social rejection of former mental patients: understanding why labels matter. *Am. J. Sociol.* **92** (6), 1461–1500 (1987).

[27] Satow, L. Skala zur Erfassung von Testverfälschung durch positive Selbstdarstellung und sozialerwünschte Antworttendenzen (SEA). Skalendokumentation Normen Sowie Fragebogen Mit Instr PSYNDEX Tests. (9006446). (2012).

[28] IBM Corp. *IBM SPSS Statistics for Mac* (IBM Corp, 2021).

[29] Gignac, G. E. & Szodorai, E. T. Effect size guidelines for individual differences researchers. *Personal Individ Differ.* **102**, 74–78 (2016).

[30] Holm, S. A simple sequentially rejective multiple test procedure. *Scand. J. Stat.* :65–70 .

[31] Holland, J. C., Kelly, B. J. & Weinberger, M. I. Why psychosocial care is difficult to integrate into routine cancer care: stigma is the elephant in the room. *J. Natl. Compr. Canc Netw.* **8** (4), 362–366 (2010). 20410331 10.6004/jnccn.2010.0028

[32] Fujisawa, D. & Hagiwara, N. Cancer stigma and its health consequences. *Curr. Breast Cancer Rep.* **7**, 143–150 (2015).

[33] Weiss, J. et al. Stigma, self-blame, and satisfaction with care among patients with lung cancer. *J. Psychosoc Oncol.* **35** (2), 166–179 (2017). 27607144 10.1080/07347332.2016.1228095

[34] Shen, M. J., Hamann, H. A., Thomas, A. J. & Ostroff, J. S. Association between patient-provider communication and lung cancer stigma. *Support Care Cancer*. **24**, 2093–2099 (2016). 26553030 10.1007/s00520-015-3014-0 PMC4805469

[35] Karadağ, E. & Özlem, U. Can Oncology Nursing Education Change the attitude of nursing students toward Cancer (Cancer Stigma)? A quasi-experimental study. *J. Basic. Clin. Health Sci.* **7** (1), 18–25 (2023).

[36] Sürücü, H. A., Topdemir, E. A., Baksi, A. & Besen, D. B. Empathic approach to reducing the negative attitudes of nursing undergraduate students towards cancer. *Nurse Educ. Today*. **105**, 105039 (2021). 34245957 10.1016/j.nedt.2021.105039

[37] Bhattacharyya, G. S., Malhotra, H., Babu, G., Vora, A. & Bhattacharyya, S. Cancer stigma related to beliefs of patients and care providers. *Ann. Oncol.* **29**, viii559 (2018).

[38] Yıldız, K. & Koç, Z. Stigmatization, discrimination and illness perception among oncology patients: a cross-sectional and correlational study. *Eur. J. Oncol. Nurs.* **54**, 102000 (2021). 34492525 10.1016/j.ejon.2021.102000

